# Murine Models of Acute Myeloid Leukemia

**DOI:** 10.3389/fonc.2022.854973

**Published:** 2022-06-08

**Authors:** Kristen J. Kurtz, Shannon E. Conneely, Madeleine O’Keefe, Katharina Wohlan, Rachel E. Rau

**Affiliations:** ^1^ Department of Pediatrics, Baylor College of Medicine, Texas Children’s Hospital, Houston, TX, United States; ^2^ Department of Molecular and Cellular Biology, Baylor College of Medicine, Houston, TX, United States

**Keywords:** acute myeliod leukemia, AML, transgenic mouse, genetically engineered mice (GEM), core binding factor acute myeliod leukemia, KMT2a (MLL) rearrangements, NUP98 fusion, patient-derived xenograft (PDX)

## Abstract

Acute myeloid leukemia (AML) is a phenotypically and genetically heterogeneous hematologic malignancy. Extensive sequencing efforts have mapped the genomic landscape of adult and pediatric AML revealing a number of biologically and prognostically relevant driver lesions. Beyond identifying recurrent genetic aberrations, it is of critical importance to fully delineate the complex mechanisms by which they contribute to the initiation and evolution of disease to ultimately facilitate the development of targeted therapies. Towards these aims, murine models of AML are indispensable research tools. The rapid evolution of genetic engineering techniques over the past 20 years has greatly advanced the use of murine models to mirror specific genetic subtypes of human AML, define cell-intrinsic and extrinsic disease mechanisms, study the interaction between co-occurring genetic lesions, and test novel therapeutic approaches. This review summarizes the mouse model systems that have been developed to recapitulate the most common genomic subtypes of AML. We will discuss the strengths and weaknesses of varying modeling strategies, highlight major discoveries emanating from these model systems, and outline future opportunities to leverage emerging technologies for mechanistic and preclinical investigations.

## 1 Introduction

Acute myeloid leukemia (AML) is a heterogeneous hematologic malignancy. The heterogeneity of AML has been understood for as long as the disease has been described and led to efforts to categorize the disease into similarly behaving subgroups (1). The earliest divisions were based on microscopic visualization. Perhaps the most well-known is the French-American-British classification system, first outlined in 1976. This system divided AML into eight subtypes based on morphology and cytochemical properties of leukemic blasts.

Advances in chromosome banding visualization techniques in the 1970s allowed for the first identification of genetic changes associated with AML. Several common, non-random cytogenetic abnormalities were found to correlate with clinical behavior, morphology, and patient outcomes with high predictability (2). These patterns included: favorable survival with inv (16) and t (8;21), increased early hemorrhage with t (15;17), and correlation of the poor-prognosis monosomy 5/7 or del 5q/7q with history of previous malignancy or carcinogenic exposures. Though not known at the time if these changes were drivers of or resulting from leukemia, these correlations provided some of the earliest evidence that the clinical heterogeneity of AML may be explained by underlying genetic heterogeneity.

It is now understood that approximately 55% of adult AML and 75% of pediatric AML is driven by a cytogenetic aberration (3–5). Presently, assessment of cytogenetic abnormalities is performed by karyotyping and fluorescent *in situ* hybridization for specific recurrent rearrangements of clinical significance (6). There are currently seven subclasses of AML that are defined by their translocations or inversions as defined by the World Health Organization (7). The most common are the same that were first identified in the 1970s. Now known as core binding factor (CBF) leukemias, those with t(8;21)(q22;q22) with resulting *RUNX1-RUNX1T1* fusion (formerly *AML1-*ETO) and inv(16)(p13q22)/t(16;16)(p13;q22) with resulting *CBFB-MYH11* fusion gene carry favorable prognoses. CBF AML is more prevalent in the pediatric population, estimated at 20-25% compared to 13% of AML in adults (4, 6). Acute promyelocytic leukemia (APL) defined by t (15;17) (q24;21) and resulting in a *PML-RARA* fusion comprises about 13% of adult and 10% of pediatric AML cases (4, 6). APL is associated with severe, sometimes life-threatening, coagulopathy at presentation. Prompt treatment with all-*trans* retinoic acid is critical to preventing early death during this high-risk time. With early recognition and treatment, survival is excellent for patients with APL, with long term remission rates as high as 85-90% (6). Monosomy 5/7 or del 5q/7q are rare and typically confer poor prognosis. Though estimated to represent only 4% of adult AML, *KMT2A* rearrangements (*KMT2A*r) are found in 20% of pediatric AML and are especially common in the infant population (4). The prognosis of *KMT2A*r AML depends on the fusion partner, over 100 of which have been identified, but is often poor (8).

In addition to cytogenetic abnormalities, extensive sequencing efforts have revealed driving gene mutations in nearly all cases of AML (6, 9–13). The mutational landscape of pediatric AML differs from that in adults, and though mutational burden increases with age overall AML has one of the lowest mutational rates amongst malignancies (13, 14). In adults the most commonly mutated genes include *fms-like tyrosine kinase* 3 (*FLT3)*, *nucleophosmin 1* (*NPM1)*, and *DNA methyltransferase 3A* (*DNMT3A)*, each occurring in approximately one third of patients (10, 12, 13, 15). While *FLT3* mutations also occur in about 30% of pediatric AML cases, *NPM1* is seen in only 10% of pediatric patients and *DNMT3A* mutations are almost never identified (13, 16–18). Along with *FLT3*, other signaling pathway-affecting mutations such as Ras pathway mutations (*NRAS, KRAS, HRAS, NF1, CBL* and *PTPN11)* and *KIT* are some of the most common in pediatric AML, and less commonly seen in adults (6, 10, 12, 13).

Many mutations have prognostic implications, and some may represent therapeutic targets. For example, *FLT3* mutations most often confer poor prognosis, depending on the allelic fraction and co-occurring mutations. Importantly, *FLT3* is a targetable tyrosine kinase receptor. The addition of *FLT3*-targeting tyrosine kinase inhibitors has significantly improved the outcome of these patients (3). *DNMT3A*, along with other methylation associated genes including *TET2*, *IDH1*, and *IDH2*, have been associated with poor prognosis in adults but are infrequently found in children (16). Each newly identified driver mutation presents the opportunity for targeted treatment with Food and Drug Administration approval of IDH inhibitors as a recent example.

Our understanding of the genomic landscape of AML continues to expand. Ongoing investigations are focused on co-occurring mutations and their effects on patient outcomes and responses to treatment. Much has been uncovered over the last several decades, and much remains to be discovered. To adequately address these remaining questions, in addition to rigorous clinical investigations of large, uniformly-treated patient populations, the availability of faithful model systems will be essential. While not all encompassing, here we will review several recent murine models selected for their relevance to human AML, with similar genetic lesions modeled, characteristic disease phenotypes, and that have advanced AML research over the last several decades. Additionally, we have included papers that highlight a spectrum of techniques used to generate murine model systems to allow for comparison of the advantages and disadvantages of each strategy that may aid in selecting the appropriate model system to address a specific research question.

## 2 History of Mouse Modeling Hematologic Malignancy

The goals of biomedical research of leukemia include predicting disease behavior to subclassify and risk stratify and developing or refining therapies to achieve the best efficacy. Animal models of human disease are vital to understanding disease pathogenesis and development of novel therapeutic strategies. The mouse (*Mus musculus*) is the most widely used animal model of human disease because of its genetic and physiologic similarity to humans. Humans and mice share approximately 80% of their genes with conservation of tissue-specific gene expression across species (19, 20). They also have remarkably similar organ systems. Other advantages to murine model systems include their small size, short lifespan, and rapid breeding, making them ideal models for scientific research. Murine hematopoiesis has been well-characterized over the years such that the similarities and differences to human hematopoiesis can be considered when creating and interpreting mouse models of leukemia.

When using murine models, it is crucial that each model be validated and evaluated for its similarities and potential differences to human disease. Some mouse strains harbor characteristic background lesions or are prone to diseases that are related to mouse biology rather than the model of human disease they are meant to represent. These details must be teased out so as not to attribute unrelated sequelae to the model of human disease. Differences in the human and murine genome as well as differences in hematopoiesis, aging and general development may impact the evolution and behavior of leukemia within the mouse model and must be considered. There are innate differences in hematopoiesis between humans and mice that may influence interpretation of mouse models. For example, mice exhibit lymphocyte predominance in the circulating leukocyte population while humans exhibit neutrophil predominance (21). There may also be subtle differences in the hematopoietic niche that affect leukemia development and progression that are unidentified and should be considered when interpreting results from mouse models.

The earliest mouse models of leukemia were created *via* exposure to external carcinogens. These included carcinogenic chemicals, irradiation, and viruses. The first transplantable leukemic mouse cell lines (L1210 and p388) were isolated from DBA/2 mice following exposure to 3-methylcholantrene (22, 23). These mouse models were highly valuable for exploring drug efficacy and developing strategies to overcome drug resistance (24). However, such chemically induced models can be inefficient and imprecise when used to recapitulate a specific malignant process and more often resulted in lymphoblastic rather than myeloid leukemia (25–27). Once the link between radiation and leukemia was established from observational studies involving individuals exposed to excessive radiation from nuclear attacks, the RF mouse model of myeloid leukemia was created *via* exposure to ionizing radiation (28). While this method effectively mirrored an actual environmental trigger and subsequent leukemic process to establish this causal relationship, there was a 6–8-month latency to leukemia onset and a low incidence of leukemia in the exposed mice, rendering it inefficient to create a robust cohort of leukemic mice for further study. Alternatively, viruses have been used to induce a more efficient animal model of leukemia. Murine leukemia viruses (MuLV) are retroviruses that have been used to induce myeloid leukemias in mice with a relatively short latency since the 1950s (29–31). Virally-induced mouse models of AML led to important discoveries of previously unknown oncogenes and the underlying pathogenesis of leukemia (32–34).

Gene editing has recently revolutionized the way in which mouse models of leukemia are generated. Advancements during the 1970-80s introduced techniques that allowed scientists to deliver engineered genetic material into the murine genome, thus creating transgenic mouse models. As advancements in high-throughput sequencing led to a wealth of new data on the genetic underpinnings of hematologic malignancy, gene editing paved the way for novel animal models of these newly defined genetic subtypes of disease. This led to more precise models that better mimicked the progression and behavior of subgroups of human leukemias. These techniques were first used to introduce proto-oncogenes under the control of a constitutively activated promoter to define their role in leukemogenesis in the 1980s (35, 36). Since then, the field of gene editing has advanced significantly to allow for a variety of constitutive and inducible models that can be integrated to more precisely recapitulate leukemogenesis.

## 3 Mouse Models of AML Fusions

Classic cytogenetic rearrangements found in AML are associated with the generation of fusion genes which demonstrate altered function compared to their wild-type components (**Table 1**). Here, we will review murine models used to study these classic fusion genes as well as newly described fusion genes not identified *via* standard cytogenetic testing.

**Table 1:** Mouse models of fusion genes.

Fusion Gene	Year	Expression	Mechanism	Phenotype	References
*RUNX1-RUNX1T1*	2006	Constitutive	Retrovirus	9a isoform: AML	(37)
	2001	Constitutive	Germline - *Mrp8* promoter (myeloid specific)	AML, T-ALL after ENU treatment	(38)
	2013	Inducible	Tet-On	MDS	(39)
	2021	Inducible	eR1-CreER^T2^	AML, MPD	(40)
*CBFB-MYH11*	2006	Inducible	Mx1-Cre	AML	(41)
***KMT2A-MLLT3***	1996	Constitutive	Germline	AML	(42)
	2000	Inducible	Lmo2-Cre	AML	(43)
	2013	Constitutive	Retrovirus	AML (Transduced LSKs > GMP)	(44)
	2016	Inducible	Retrovirus	AML	(45)
*MLL-*PTD	2012	Constitutive	Germline	AML (if *FLT3*-ITD mutated)	(46)
***KMT2A-MLLT1***	2013	Inducible	CreER	ALL	(47)
	2014	Inducible	Tet-On	ALL	(48)
* NUP98-NSD1*	2007	Constitutive	Retrovirus	AML	(49)
	2014	Constitutive	Retrovirus	AML (if *FLT3*-ITD mutated)	(50)
	2020	Constitutive	Retrovirus	AML	(51)
Other *NUP98* fusions	2020	Inducible	Tet-On Retrovirus	AML	(52)
*CBFA2T3-GLIS2*	2019	Inducible	Tet-On	AML	(53)
*PML-RARA*	1997	Constitutive	Germline – *Ctsg* promoter (myeloid specific)	AML – long latency	(54)
	1999	Constitutive	Retrovirus	Differentiation blockade, enhanced self-renewal	(55)
	2003	Constitutive	Germline – *Mrp8* promoter (myeloid specific)	AML	(56)

AML, acute myeloid leukemia; ENU, N-ethyl-N-nitrosurea; GMP, granulocyte-macrophage progenitor; ITD, internal tandem duplication; LSK, Lin-Sca1+Kit^+^; MDS, myelodysplastic syndrome; MPD, myeloproliferative disease; PTD, partial tandem duplication; T-ALL, T-acute lymphoblastic leukemia.

### 3.1 Core Binding Factor Leukemias

CBF AML encompasses patients with t(8;21) or inv(16) cytogenetic rearrangements or their associated fusion genes, *RUNX1-RUNX1T1* and *CBFB-MYH11*, respectively (57). The chimeric proteins which result from CBF AML fusion genes function as dominant negative inhibitors of the CBF transcription factor, composed of RUNX1 and CBFβ, which are essential for normal myeloid cell development. The CBF AML fusion genes are associated with a favorable prognosis and share a common pathogenic mechanism, though *RUNX1-RUNX1T1* and *CBFB-MYH11* driven leukemias are distinct from one another.

Pioneering work from the labs of Drs. Dong Er Zhang, Nancy Speck and others have generated various murine model systems that have enhanced our understanding of this common AML fusion. Initial attempts to model t (8;21) AML utilized a germline knock-in of the *RUNX1*-*RUNX1T1* fusion gene (58). However, embryonic expression of RUNX1-RUNX1T1proved to be embryonic lethal due to central nervous system hemorrhage and failed hematopoiesis. While this method failed to create a model of t (8;21) leukemia, these early studies helped to establish the role of *RUNX1-RUNX1T1* as an inhibitor of normal RUNX1 function and highlighted the need for alternative murine models with delayed *RUNX1-RUNX1T1* expression.

**Figure 1:**
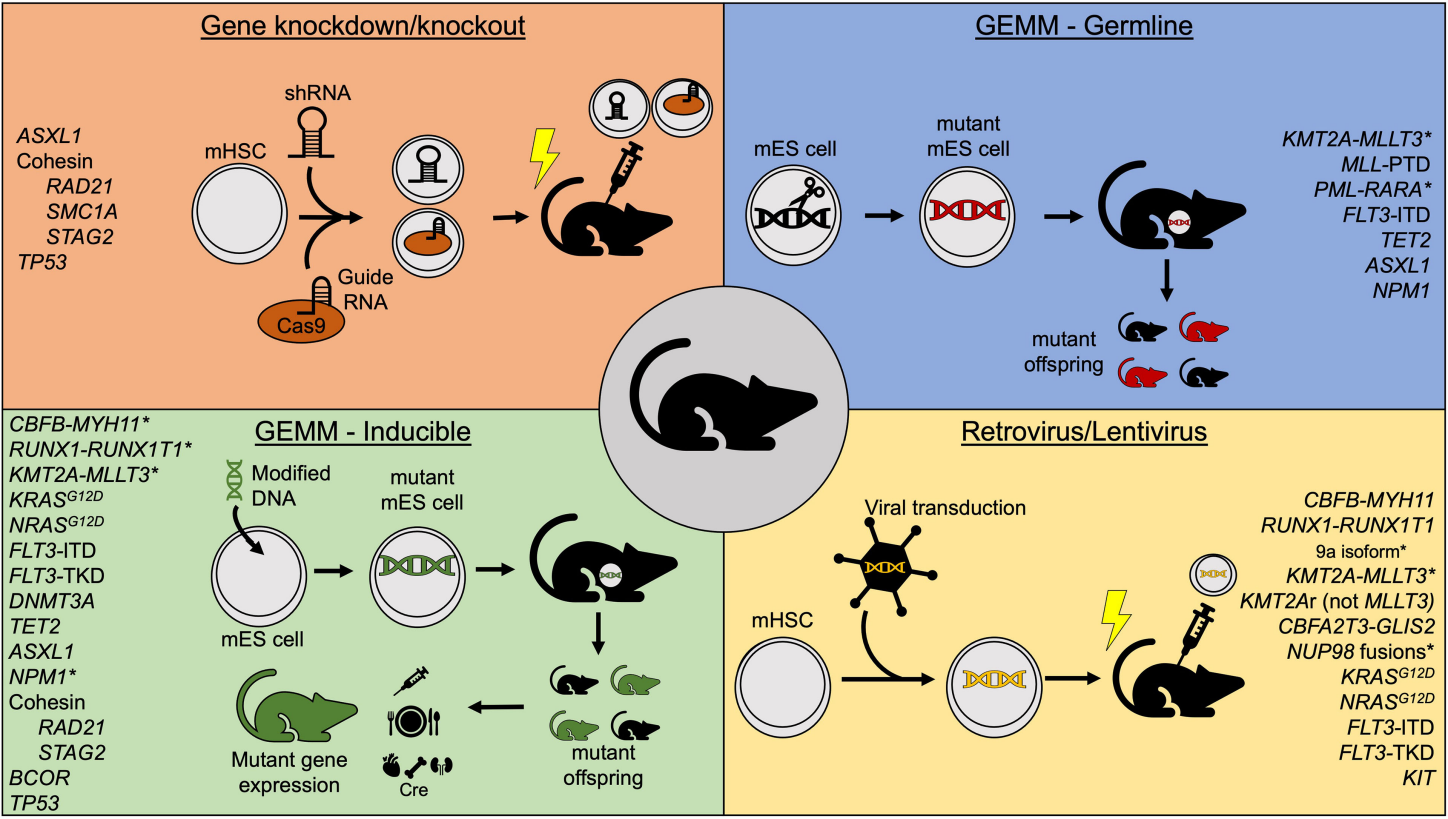
Summary of commonly used mouse model methods to study acute myeloid leukemia. *Indicates models that independently generate leukemia without cooperating mutations. GEMM, Genetically-engineered mouse model; mHSC, murine hematopoietic stem cell; mES, murine embryonic stem cell; shRNA, short hairpin RNA.

Current murine models of t (8;21) AML rely on delayed introduction or expression of the *RUNX1-RUNX1T1* fusion through various means (**Figure 1**). One method utilizes retroviral transduction to introduce constitutive expression of *RUNX1-RUNX1T1* from a retroviral vector incorporated into the DNA of a cell of interest. Retroviral expression of the full length 752 amino acid protein in murine hematopoietic stem cells (HSCs) failed to produce leukemia or signs of altered hematopoiesis when transplanted into lethally irradiated recipient mice, however, and led to the conclusion that *RUNX1*-*RUNX1T1* alone is insufficient for leukemogenesis (59). Yan et al. subsequently identified a splice variant which generates a 575 amino acid protein, termed RUNX1-RUNX1T19a, that is variably expressed in human AML and generates leukemia when transduced into murine fetal liver cells (37). All mice transplanted with RUNX1-RUNX1T19a expressing cells developed leukemia within 16 weeks of transplant. As this is the only retroviral model to establish a t(8;21)-like AML without the addition of cooperating mutations, it is commonly used for *in vivo* modeling of *RUNX1-RUNX1T1* driven disease, though the full-length construct remains the preferred model for *in vitro* experiments.

Transgenic models in which *RUNX1-RUNX1T1* is transcribed in mice only in the presence of specific drivers have also been developed. One example is the *MRP8-AE* mouse in which *RUNX1-RUNX1T1* is expressed in myeloid cells under control of the *MRP8* promoter (38). Consistent with results from retroviral models, these mice remain healthy during their lifetime unless secondary mutations are induced with the alkylating agent *N*-ethyl-*N*-nitrosourea. However, only 55% of leukemia generated *via* this method was AML, while the remaining 45% was T-cell acute leukemia. A recent study published by Abdallah et al. used a model in which the *RUNX1-RUNX1T1* downstream of a lox-stop-lox cassette was knocked-in to the ROSA26 locus such that Cre mediated recombination leads to excision of the stop codon and induced expression of the knocked-in fusion gene in HSCs at varying ages (40). They demonstrated that earlier expression of *RUNX1-RUNX1T1*, as early as postnatal day 3, resulted in a higher AML penetrance and lower incidence of non-AML disease compared to mice where *RUNX1-RUNX1T1* expression was induced at 8-16 weeks of age. This is the only transgenic model and full-length isoform to consistently produce AML in the absence of other mutations. An alternative model uses a ‘tet-On’ method to express *RUNX1-RUNX1T1* [ROSA26-iM2-tetrO-GFP/TgPtet-AML1-ETO (*R26/AE*)] (39). *R26/AE* mice that also carry a reverse tetracycline transactivator *(rtTa*) gene will express *RUNX1-RUNX1T1* from a tetracycline-responsive promoter when exposed to doxycycline in their food or water. However, transplantation of bone marrow from *R26/AE rtTa^+/-^* mice into wild-type recipients is required to isolate expression to hematopoietic cells. Prolonged expression of *RUNX1-RUNX1T1* in this system led to expansion of myeloid progenitor cells and produced myelodysplastic syndrome (MDS) with peripheral blasts without overt leukemia.

Similar to the effects of *RUNX1-RUNX1T1* expression in embryos, germline knock-in of *CBFB-MYH11* is embryonic lethal due to multiple fetal hemorrhages and failed hematopoiesis (60). However, early models showed that induced expression of *CBFB-MYH11* in the hematopoietic system could independently induce leukemia in mice. This was robustly demonstrated by *Kuo* et al. who developed a conditional knock-in of the *Cbfb-Myh11* gene *(CBFB^56M^)* (41). Following induction of Cre recombinase expression in *Mx1-Cre^+^ CBFB^56M^* mice with polyinosinic: polycytidilic acid (pIpC), the fusion gene is knocked in to the native *Cbfb* locus. Treated mice develop AML 11-21 weeks following the fusion gene restoration. This model has been used extensively to characterize inv (16) AML, cooperating mutations, and essential partners in leukemogenesis.

### 3.2 *KMT2A* Fusions

*KMT2A*r AML poses a particular challenge for mouse modeling strategies, as over 100 different fusion genes are known to partner with *KMT2A* as a result of 11q23 rearrangements (61). Over 90% of *KMT2A*r AML is caused by 9 specific fusions, and the frequency of these fusions varies with patient age. Thus, established murine models represent only the most common *KMT2A* fusions.

*KMT2A-MLLT3* (formerly *MLL-AF9)* fusions are the most common KMT2A rearrangement in AML and account for 25-40% of *KMT2A*r AML across all age groups (61). Murine models have all demonstrated that *KMT2A-MLLT3* is a potent oncogene with the ability to induce leukemic transformation. Early studies produced genetically engineered germline *Kmt2a-MLLT3* mutants in which the fusion is expressed in all cells throughout development (42). Although this model did lead to development of AML, or less commonly acute lymphoid leukemia, there are known effects of *Kmt2a* haploinsufficiency during murine development that cannot be controlled using this method, such as anemia, thrombocytopenia, and decreased B-cells (62). Thus, a *Cre-loxP* system was introduced such that intrachromosomal recombination of *Kmt2a and Mllt3* could be induced *via* Cre-mediated recombination (43). Although effective in generating AML when *Kmt2a-Mllt3* recombination was induced in primitive progenitor cells *via* progenitor-specific Cre, *Lmo2-Cre*, off-target expression of Cre recombinases poses concerns regarding specificity for cellular populations of interest (63). This is particularly true in *KMT2A*r AML, where cell of origin can dictate the resultant type of leukemia and gene expression pattern (44).

To restrict expression of *KMT2A-MLLT3* to specific stem and progenitor populations and study the differing effects on each, retroviral transduction of isolated bone marrow stem/progenitor cells with *KMT2A-MLLT3* followed by transplantation has become a popular model. Lineage^-^Sca-1^+^Kit^+^ (LSK) cells expressing *KMT2A-MLLT3* are more potent inducers of leukemic transformation than *KMT2A-MLLT3* expressing granulocyte-macrophage progenitors (44). Interestingly, leukemia generated from transduced LSK cells is also more chemoresistant than that originating from granulocyte-macrophage progenitors, is more highly methylated, and carries a gene expression signature associated with poor prognosis in patients with AML (44). Selection of appropriate cell population for retroviral transduction and attendant gene expression is therefore integral to the interpretation of results from *KMT2A*r models and relating them to human AML. One concern regarding retroviral transduction is the possibility of supraphysiologic expression of *KMT2A-MLLT3* which may significantly impact study results. Recently, doxycycline-inducible genetic *KMT2A-MLLT3* models have helped to address these concerns. These models demonstrate dose-dependent expression of *KMT2A-MLLT3* resulting in 10-20-fold lower protein amounts compared to retroviral transduction models, allowing improved control over fusion gene expression (45). As with retroviral models, cells of interest can be isolated and transplanted into recipients, though expression of *KMT2A-MLLT3* can be induced before or after transplantation. These studies have confirmed effects of *KMT2A-MLLT3* expression on different stem and progenitor populations identified from studies using retroviral transduction and demonstrate their utility in future mouse modeling of *KMT2A-MLLT3* AML.

As *KMT2A-MLLT3* is the most common *KMT2A*r fusion gene in AML, it is also the most studied. After *KMT2A-MLLT3*, the next most common fusion partners are *MLLT10, ELL, AFDN, MLLT1*, and *SEPT6* (61). Murine models of these *KMT2A* fusions depend primarily on retroviral transduction of murine HSCs or human hematopoietic cells isolated from cord blood (64–66). As an alternative, immortalized murine cells lines expressing *KMT2A-MLL10* and *KMT2A-ELL* have been created which engraft into syngeneic recipients and cause disease (67, 68). In addition to recurrent *KMT2A* fusions, partial tandem duplications (PTD) of *KMT2A* are also common in adult AML while nearly absent from infant and pediatric cases (61). A germline knock-in model of *Kmt2a*-PTD exists but requires cooperating mutations, such as a *Flt3* internal tandem duplication (ITD), to produce disease (46). Transgenic mouse models of *KMT2A-MLLT1* have also been created and characterized including one which utilizes Cre recombination to induce expression of *KMT2A-MLLT1* and the other with doxycycline-inducible expression of *KMT2A-MLLT1* (47, 69). However, *KMT2A-MLLT1* fusions are much more common in ALL and occur rarely in AML, limiting the use of these models in AML research. Finally, CRISPR/Cas9-mediated genome editing has been used to create a model of *Kmt2a-Mllt3* AML *via* dual single-guide RNAs simultaneous targeting the breakpoint cluster region of *Kmt2a* and *Mllt3* (70). Unique to *KMT2A*r AML, close attention must be paid to the model system and cell of origin in studying *KMT2A*r disease, as similar *KMT2A* fusions can be found across hematologic malignancies, and selection of methods will have profound effects on the resulting disease.

The development of these varying murine models of *KMT2A*r AML have helped identify potential novel therapeutic strategies for this poor prognosis subset of AML. These include Dr. Katherine Bernt and colleagues’ identification of the histone 3, lysine 79 methylase, DOT1L, as a critical dependency and therapeutic vulnerability in *KMT2A*r AML (48). Other investigations have revealed preclinical efficacy of BET bromodomain inhibitors in *KMT2A*r AML (71). Perhaps the most exciting, pioneering work by Dr. Jolanta Grembecka and colleagues has led to the development and validation of inhibitors of the MENIN-MLL interaction as highly promising agents for the treatment of *KMT2A*r leukemias (72). Results from these seminal investigations have led to early phase clinical trials and ultimately could result in meaningful improvements in outcomes for patients with *KMT2A*r AML.

### 3.3 *NUP98* Fusions

While relatively uncommon in AML, *NUP98* gene fusions carry a dismal prognosis even with stem cell transplant. Thus, establishment of mouse models is a high priority to identify novel treatments for this chemoresistant AML. Retroviral models of NUP98 fusions have served as the predominant mouse models to date. In 2007 Wang et al. (49) showed that retroviral transduction of *NUP98-NSD1*, the most common *NUP98* fusion in pediatric AML, into murine hematopoietic progenitors followed by transplantation independently produced AML with an average survival of 126 days post-transplant (68). Mohanty et al. created a similar retroviral model of *NUP98-NSD1* and demonstrated significant disease acceleration when *NRAS* ^G12D^ is co-expressed. Furthermore, the authors found that upregulation of *Hox* genes, specifically *Hoxa7, Hoxa9*, and *Hoxa10* was a major contributor to disease development, while other studies have demonstrated a dependence on interaction between NUP98-NSD1 and SMARCA5 for leukemic transformation (51, 73). A separate study introduced three different AML-specific *NUP98* fusions (*NUP98-NSD1, NUP98-KDM5A*, and *NUP98-DDX10)* into murine fetal liver cells *via* retrovirus with doxycycline-inducible expression of the fusion gene tagged with GFP (52). Following transplantation into recipient mice and induction with doxycycline, all 3 tested *NUP98* fusions rapidly produced leukemia. This also identified *cyclin-dependent kinase 6* (*CDK6)* as an integral disease driver and that pharmacologic inhibition of *CDK6* could prolong survival in these mice. Interestingly, *CDK6* is also an important driver in *KMT2A*r AML (74). Other murine-based models of *NUP98* fusions suggest that NUP98 fusion proteins interact with MLL1, suggesting a common link between these two genetic AML subtypes (75). *Ex vivo* drug sensitivity assays have also been performed using retroviral transduction models, identifying BRD2/4 inhibitors, topoisomerase II inhibitors, and gemcitabine as effective in *NUP98-KDM5A* AML (76). Finally, Heikamp et al. recently used retroviral transduction of murine hematopoietic cells with *NUP98-HOXA9* or *NUP98-KDM5A* followed by transplantation to generate pre-clinical models which they used to define MENIN-MLL1 inhibition as a potential new treatment strategy for this refractory disease (77). It is important to note that, like *KMT2A*r AML, the cell of origin of gene expression significantly impacts disease phenotype. *NUP98-KDM5A* is often associated with acute erythroid or megakaryoblastic leukemia in patients, but when expressed in mouse HSPCs then transplanted into syngeneic recipients leads to an AML-like myeloid phenotype with blasts expressing the mature myleoid markers, Cd11b and Gr1in transplant models (52, 78).

### 3.4 *CBFA2T3-GLIS2*

Lopez et al. (53) developed a novel transgenic model using tetracycline-inducible expression of CBFA2T3-GLIS2 in which nearly all mice developed a lethal hematologic malignancy, 20% of which displayed megakaryoblastic markers with a disease latency of 164 days, whereas the remaining mice displayed heterogeneous non-megakaryoblastic immunophenotypes with a significantly longer disease latency (79). As this fusion is predominantly found in pediatric AML, the authors sought to investigate the role of developmental stage on leukemia onset. They found that transplantation of fetal liver cells from their inducible model led to a shorter disease latency with a predominant megakaryoblastic population compared to mice transplanted with bone marrow from adult mice, demonstrating that developmental stage significantly impacts disease phenotype. The Lopez et al. (53) transgenic model was a substantial technical advancement as prior to its description, murine models had been largely limited to patient-derived xenografts (PDXs) (80).

### 3.5 *PML-RARA*

APL, characterized by the *PML-RARA* gene fusion, is a unique disease entity that requires vastly different treatment than standard AML and has significantly superior outcomes. Murine models have been integral to understanding the pathophysiology of APL and critical tools for the development of current targeted treatment strategies. Retroviral transduction models used to express *PML-RARA* in hematopoietic cells demonstrate increased cellular self-renewal and differentiation blockade but fail to generate leukemia when transplanted into recipient mice (55). Transgenic models have therefore become preferred with multiple models available, each with slightly different features. The earliest transgenic models expressed *PML-RARA* under control of sequences that regulate the expression of the human cathepsin G gene in myeloid cells, though leukemia penetrance was low at 30% and disease latency was prolonged (54). This model was improved upon with *PML-RARA* under control of the murine cathepsin G (*MRP8*) promoter, yielding higher expression of the fusion product and 90% leukemia penetrance with continued prolonged disease latency (56). This *MRP8-PML/RARA* model is the predominant model currently used in APL research. However, while AML does occur, differentiation arrest characteristic of APL varies and is less pronounced than in human correlates. Prior efforts have attempted to improve on this model by selectively expressing the fusion in promyelocytes to mimic the suspected origin cell population in human APL but fail to induce leukemia or demonstrate enhanced self-renewal properties of *PML-RARA-*expressing cells despite a distinct gene expression signature (81).

## 4 Mutations That Activate Signal Transduction Pathways

Mutation profiling studies have subsequently identified activating mutations in genes involved in signal transduction pathways as commonly mutated in both adult and pediatric AML and key targetable lesions (**Table 2**). These mutations generally occur at hotspot locations within the gene and are thus ideal to recapitulate with murine models that mimic activation of these pathways. Here we will review the murine models currently used to study these activating mutations.

**Table 2:** Mouse models of signal transduction pathways.

Gene Mutation	Year	Expression	Mechanism	Phenotype	References
*Kras^G12D^*	2004	Inducible	Mx1-Cre	MPD	(82)
	2009	Inducible	Mx1-Cre then transplant	T-ALL/lymphoma, JMML	(83)
* +Dnmt3a^-/-^*	2015	Inducible	Mx1-Cre	MPD, AML	(84)
* +PML-RARA*	2006	Inducible	Mx1-Cre	APL	(85)
* +RUNX-RUNX1T1*	2014	Constitutive	Retrovirus	AML	(86)
*Nras^G12D^*	2013	Inducible	Mx1-Cre	MPD	(87)
* +Dnmt3a^+/-^*	2015	Inducible	Mx1-Cre	MPD/AML	(84)
* +RUNX1-RUNX1T1*	2014	Constitutive	Retrovirus	AML	(88)
*KIT*	2011	Constitutive	Retrovirus	MPD	(87)
* +CBFB-MYH11*	2012	Constitutive	Retrovirus	Accelerated AML	(89)
* +RUNX1-RUNX1T1*	2011	Inducible	Retrovirus	AML	(87)
*FLT3-*ITD	2005	Constitutive	Retrovirus	MPD	(90)
	2005	Constitutive	Germline – *Vav* promoter (hematopoietic specific)	MPD	(91)
	2007	Constitutive	Germline	CMML (AML if combined with *KMT2A*-PTD, *Npm1c*, *Dnmt3a, RUNX1-RUNX1T1)*	(46, 92–95)
	2008	Inducible	Mx1-Cre	MPD (AML if combined with *Npm1c* or *WT1*)	(96–98)
*FLT3-*TKD	2005	Constitutive	Retrovirus	ALL	(90)
D835Y	2014	Constitutive	Germline	MPD	(99)

AML, acute myeloid leukemia; APL, acute promyelocytic leukemia; CMML, chronic myelomonocytic leukemia; JMML, juvenile myelomonocytic leukemia; ITD, internal tandem duplication; MPD, myeloproliferative disease; T-ALL, T-acute lymphoblastic leukemia; TKD, tyrosine kinase domain.

### 4.1 Ras Pathway

The Rat Sarcoma virus (Ras) family of genes represents critical regulators of cell proliferation and differentiation and is mutated across a variety of cancers. Activating mutations in Ras family genes including *NRAS, KRAS, NF1, PTPN11* and *CBL* occur in up to 50% of pediatric AML but are less common in adult AML. The predominant model used to study *KRAS* mutations is used in both hematologic and solid malignancies and was originally developed by Dr. Erica Jackson and colleagues as a model for lung cancer (100). This model has lox-stop-lox *Kras^G12D^* knocked into the native *Kras* locus leading to expression of mutant *Kras* following Cre recombination. When combined with tissue-specific Cre drivers, the mutation is expressed only in the tissue of interest. Induction of *Kras^G12D^* expression in the hematopoietic system *via Mx1-Cre* leads to a rapid and highly penetrant myeloproliferative disease (MPD) but not overt AML (82). Concurrent homozygous loss of *Dnmt3a* cooperates with *Kras^G12D^* to accelerate disease progression and leads to development of AML in approximately 30% of mice, though heterozygous loss of *Dnmt3a* had no effect (84). *Kras^G12D^* also cooperates with *PML-RARA* to produce malignancy consistent with APL with a short latency of only 37 days (85). Despite the ability to target specific cell populations with tissue-specific Cre drivers, this model has also been used in transplantation experiments where induced bone marrow from primary transgenic mice is transplanted into recipient mice along with supportive bone marrow (83). Interestingly, transplant recipients predominantly develop T-cell leukemia or lymphoma and occasionally juvenile myelomonocytic leukemia in contrast to the MPD described above, thus limiting the use of transplant models to study AML biology (83).

*NRAS* is another Ras pathway gene that is commonly mutated in AML. As with *KRAS* mutations, *NRAS* mutations are activating mutations that have been modeled using genetic mouse models. When expressed in the hematopoietic system, *Nras^G12D^* alone induces a MPD similar to *Kras^G12D^* but with significantly longer disease latency and lower penetrance (88). However, *Nras^G12D^* cooperates with heterozygous loss of *Dnmt3a* to promote AML development in one third of induced mice (84). As *DNMT3A* mutations are predominantly heterozygous in human disease, this may represent a more biologically relevant model of AML.

Retroviral transduction models of Ras mutants have also been employed to study cooperating mutations in AML. Zhao et al. used co-transduction of *KRAS^G12D^* or *NRAS^G12D^* and *RUNX1-RUNX1T1* followed by transplantation to demonstrate cooperation between activating Ras mutations and *RUNX1-RUNX1T1* in leukemogenesis (86). Retroviral expression of either *KRAS^G12D^* or *NRAS^G12D^* alone did not induce leukemia or an identified MPD, yet both cooperated with *RUNX1-RUNX1T1* to accelerate development of AML with *NRAS* serving as a more potent inducer of disease in this context. Retroviral expression of *NRAS^G12D^* has also been shown to cooperate with loss of *Dnmt3a* in mouse models to generate AML (101). Retroviral expression of Ras mutants may therefore serve as a reasonable method when transplantation models using AML-specific drivers are preferred.

### 4.2 *KIT*

Tyrosine protein kinase KIT, encoded by the gene *KIT (formerly c-KIT)*, is a proto-oncogene which plays a critical role in signaling pathways that promote cellular proliferation, particularly within HSCs. Activating mutations in *KIT* have been found at several gene loci in patients with AML, and such mutations occur in nearly 40% of patients with CBF AML (102). These mutations are predominantly found in exons 8 and 17 with amino acids D816 and N822 serving as recurrent mutation hotspots. As with other tyrosine kinase activating mutations, studies have shown that activating mutations in *KIT* are not sufficient to cause leukemia but cooperate with other driver mutations to transform leukemic cells. Retroviral transduction of the two recurrent exon 17 *KIT* mutant genes into *CBFB-MYH11-*expressing bone marrow cells shortened survival of transplant recipients compared to those which expressed wild-type *KIT* (89). In a model of t (8;21) AML in which retrovirus expression *KIT* N822 and *RUNX1-RUNX1T1* were co-transduced, expression of the *KIT* mutant alone led to MPD, but overt leukemia was observed when *KIT^N822^* was expressed in conjunction with *RUNX1-RUNX1T1* (87).

### 4.3 *FLT3*

*FLT3* is a commonly mutated gene in AML, leading this receptor tyrosine kinase to be constitutively active driving uninhibited cell growth. Internal tandem duplications (*FLT3*-ITD) of the juxtamembrane domain encompass a majority of *FLT3* mutations, though activating mutations in the tyrosine kinase domain (*FLT3*-TKD) also occur (10, 13). Early retroviral transduction models of both *FLT3*-ITD and *FLT3*-TKD mutations revealed that expression of these activating mutations lead to distinctly different disease phenotypes (90). Transplantation of *FLT3-*ITD expressing bone marrow led to MPD, whereas transplantation of *FLT3-*TKD expressing cells led to a lymphoid disease with longer latency, consistent with the finding that TKD mutations are more common in acute lymphoblastic leukemia than AML (103). In addition, these studies revealed that *FLT3*-ITD mutations, and not *FLT3*-TKD, lead to activation of STAT5 signaling.

Several transgenic models of *FLT3*-ITD have been developed, each with slightly different advantages, though they predominantly produce MPD. First, a transgenic model expressing *FLT3-*ITD under the control of the hematopoietic-specific *Vav* promoter was created by the lab of Dr. D. Gary Gilliland which developed MPD at 6-12 months (91). Subsequently, the same group developed a model in which a humanized ITD is knocked into the native murine *Flt3* gene (92). This model minimizes the effects of gene overexpression on disease phenotype inherent to retroviral transduction or use of heterologous promoters, as expression levels of FLT3 in both wild-type and mutant forms have a significant impact on disease. In the absence of cooperating mutations, this model leads to the development of chronic myelomonocytic leukemia (CMML) and not AML. However, when used in combination with other common AML lesions, such as *KMT2A-*PTD, *Npm1c* mutation, *Dnmt3a* deletion, and *RUNX1-RUNX1T1*, expression of *Flt3-*ITD is capable of producing AML, though with relatively long latency (46, 93–95). A similar model in which an 18-base pair ITD was knocked into the juxtamembrane domain of native *Flt3* gene was also developed by Dr. Li in the lab of Dr. Donald Small. This model is characterized by the development of MPD with a median survival of 10 months (96), and cooperates with other common AML mutations, including *NPM1c* and mutant *Wt1*, to generate AML or accelerate the MPD disease process (97, 98).

*FLT3*-TKD mutations also occur in AML but are less common and not as strongly associated with prognosis compared to ITD mutations. Plasmids conferring the expression of various *FLT3*-TKD mutants have been developed but are predominantly used to transform cell lines, and murine models of *FLT3*-TKD mutants are lacking (99, 104, 105). Dr. Emily Bailey and colleagues previously generated a knock-in of the most common TKD mutant, D835Y, which developed predominantly MPD but with a longer latency than ITD models (106). Thus, new murine models which cover the spectrum of relevant *FLT3* aberrations are needed to adequately study this common mutation in AML.

## 5 Mutations That Affect Transcription Factors or Epigenetic Modifiers

Genomic landscape studies have revealed critical epigenetic modifiers that are frequently mutated in myeloid malignancies (**Table 3**). Here we will review data from some of the best characterized murine models of these epigenetic modifier mutations.

**Table 3:** Mouse models of epigenetic regulators.

Genemutation	Year	Expression	Mechanism	Phenotype	References
*Dnmt3a (KO)*	2011	Inducible	Mx1-Cre	Enhanced self-renewal	(107)
	2015	Inducible	Mx1-Cre	MDS, AML, ALL	(101)
	2015	Inducible	Mx1-Cre	MDS, AML, MDS/MPN	(108)
*Dnmt3a^R882^*	2016	Inducible	Mx1-Cre	HSPC expansion, myeloid bias, AML when combined with FLT3+Npm1c	(109)
	2019	Inducible	Mx1-Cre	AML on serial transplant co-expressed with Npm1c	(110)
*Dnmt3a (KO)* * + Flt3*	2017	Inducible	Mx1-Cre	ALL, AML	(94)
		Inducible	Retrovirus		
*Dnmt3a (KO)* * + Tet2*	2016	Inducible	Mx1-Cre	ALL, AML	(111)
		Constitutive			
*Dnmt3a (KO)* * + Idh2*	2020	Inducible	Mx1-Cre	AML	(112)
		Inducible	Retrovirus		
*Tet2 (KO)*	2011	Inducible	Mx1-Cre	CMML-like	(113, 114)
	2011	Constitutive		Increased BM cellularity and HSPC expansion	(115)
	2011	Constitutive		CMML, MPN, MDS (low penetrance)	(116)
	2012	Constitutive		Mild myeloproliferation	(117)
*Tet2 (KO)* * + NRAS*	2018	Inducible	Mx1-Cre	CMML	(118)
		Inducible	Mx1-Cre		
*Tet2 (KO)* * + KIT*	2018	Constitutive		MPN	(119)
		Inducible	Mx1-Cre		
*Asxl1 (KO)*	2013	Inducible	Mx1- and Vav- Cre	MDS	(120)
	2014	Constitutive		MDS, some CMML	(121)
*Asxl1 (mut)*	2018	Inducible	Vav-Cre	Mild anemia	(122)
	2021	Constitutive		Mild splenomegaly	(123)
*Smc3*	2015	Inducible	Mx1-Cre	None (shortened latency of AML when added to Flt3-ITD)	(124)
*Smc1a*	2015	Inducible	rtTAs	MPN-like	(125)
*Bcor*	2017	Inducible	CreER	T-ALL	(126)
	2019	Inducible	CreER	AML if combined with *Kras^G12D^*	(127)
	2021	Inducible	Mx1-Cre	(erythroid) AML	(128)

AML, acute myeloid leukemia; BM, bone marrow; CMML, chronic myelomonocytic leukemia; HSPC, hematopoietic stem/progenitor cell; ITD, internal tandem duplication; KO, knock-out; MDS, myelodysplastic syndrome; MPD, myeloproliferative disease; MPN, myeloproliferative neoplasm; PTD, partial tandem duplication; T-ALL, T-acute lymphoblastic leukemia.

### 5.1 *DNMT3A*

DNA methyltransferase 3A (DNMT3A) is a *de novo* DNA methyltransferase that methylates cytosine moieties of CpG dinucleotides (129). HSCs frequently acquire *DNMT3A* mutations which act as pre-leukemic lesions and in turn lead to clonal hematopoiesis which, in some cases, ultimately progresses to leukemia. Up to 22% of adult *de novo* AML cases (130, 131) and 10% of MDS (107, 132) harbor somatic mutations in *DNMT3A*, most occurring at arginine 882 (R882) in the DNMT3A methyltransferase domain. Since *DNMT3A* variants were first reported in AML in 2010, a variety of mouse modeling techniques have been used to clarify the role of *DNMT3A* in HSCs and the precise role that loss of function of *DNMT3A* plays in leukemogenesis.

In 2011, the lab of Dr. Margaret Goodell reported the effects of *Dnmt3a* deficiency *in vivo* using an inducible *Dnmt3a* knock-out (KO) murine model (133). Transgenic animals carrying hematopoietic tissue-specific *Mx1-Cre* were crossed with mice carrying *loxP*-flanked copies of *Dnmt3a*, and *Dnmt3a* loss was then induced *via* serial intraperitoneal injections of pIpC. In competitive transplant experiments, equal parts purified HSCs from non-induced *Dnmt3a^fl/fl^* and wild-type mice were transplanted into primary recipients followed by induced deletion of *Dnmt3a*. They found no difference in the represented proportion of HSCs before or after *Dnmt3a* deletion was induced in primary recipients. However, in secondary competitive transplants, there was a significant increase in *Dnmt3a-*null HSCs compared to wild-type, demonstrating that loss of *Dnmt3a* led to enhanced stem cell self-renewal. No mice developed MPD or overt leukemia suggesting that additional cooperating mutations are necessary for leukemogenesis.

Similar inducible models have since been used in non-competitive transplants resulting in a variety of hematologic malignancies, including MDS, AML, primary myelofibrosis, and T- and B-cell acute lymphoblastic lymphoma. Dr. Allison Mayle and colleagues demonstrated that with non-competitive transplantation of *Dnmt3a*-null bone marrow, all transplanted mice died between 200- and 400-days post-transplant from a variety of hematologic malignancies (101). Challen et al. reported similar findings of bone marrow failure resulting in death with 100% penetrance following transplantation of *Dnmt3a*-null whole bone marrow into sublethally irradiated mice (134). The majority of moribund mice met diagnostic criteria for MDS with 2/15 mice developing frank AML and 4/25 developing an intermediate MDS/myeloproliferative neoplasm. This inducible *Dnmt3a* KO model has also been combined with a variety of murine models harboring genetic lesions of genes that are commonly co-mutated in human *DNMT3A*-mutant AML, including *FLT3*, *TET2*, *IDH2*, and *KIT*, identifying critical cooperative mechanisms that drive leukemogenesis (94, 108, 111, 112).

In AML, the most common *DNMT3A* mutation affects amino acid R882 (*DNMT3A^R882^*) in the methyltransferase domain. *DNMT3A^R882^* encodes a mutant protein that is hypomorphic and exerts a dominant negative effect, interfering with wild-type *DNMT3A* function resulting in severely reduced cellular methyltransferase activity (135, 136). Several inducible models have been created to specifically replicate *DNMT3A^R882^* and elucidate the precise role of this dominant negative acting mutant in leukemogenesis. Dr. Olga Guryanova and colleagues utilized a model in which mutant *Dnmt3a^R878H^* (the mouse homolog to *DNMT3A^R882H^*) is expressed from the endogenous *Dnmt3a* locus. To do so, they replaced endogenous *Dnmt3a* exon 23 with a *Lox-Stop-Lox* cassette followed by exon 23 and 24 with the point mutation affecting amino acid R878. In this model, prior to Cre recombination, the modified allele functions as a null allele, and after Cre recombination leads to expression of the mutant mRNA and protein. When *Dnmt3a^R878^* is conditionally expressed in the hematopoietic system by induction of the hematopoietic-specific *Mx-Cre* by pIpC injections, hematopoietic stem/progenitor cell expansion and myeloid bias in differentiation were observed, but no overt leukemia (109). They were able to create a fully penetrant and robust model of AML *via* co-expression of *Dnmt3a^R878H^*, *Flt3^ITD^* and *Npm1^c^*. The lab of Dr. Jennifer Trowbridge recently created a similar conditional model of *Dnmt3a^R878H^* (110). However, an advantage of the Trowbridge model over that used by Guryanova is that prior to Cre-mediated recombination, wild-type *Dnmt3a* is expressed from the modified allele whereas the modified allele in the Guryanova model is null prior to recombination. This strategy eliminates the possible confounding impact of constitutive haploinsufficiency of *Dnmt3a* in the hematopoietic system prior to recombination.

Our group recently used CRISPR/Cas9-mediated gene editing of murine embryonic stem cells to create constitutive models germline *Dnmt3a* lesions that have been previously described in patients with the rare overgrowth, intellectual disability syndrome, Tatton-Brown-Rahman syndrome. We found that each model recapitulated the distinct growth, behavioral and hematologic phenotypes observed in their human counterparts including increased risk of hematologic malignancy (137). Dr. Amanda Smith in the lab of Dr. Timothy Ley similarly found that a germline *Dnmt3a^R878^* model also recapitulated the features of TBRS including risk of hematologic malignancy development (138). These and additional models that recapitulate specific point mutations observed in human disease will help us establish the connection between alterations in *DNMT3A* function and pathogenesis.

Further development of mouse models that utilize inducible gene deletion strategies to incorporate mutations that are known to co-occur with *DNMT3A* in human disease will help to further define the role of DNMT3A in leukemic transformation and more closely mirror the natural disease process.

### 5.2 *TET2*

*TET2* is a member of the TET family of proteins with dioxygenase enzymatic activity resulting in oxidation of the methyl group at the 5-position of cytosine. The precise effect of *TET2*’s action has not been proven, but it is hypothesized that this modified locus prevents *DNMT1*-mediated methylation during DNA replication, thus leading to passive loss of DNA methylation. Loss of function *TET2* mutations are among the most common drivers of clonal hematopoiesis and have been detected in 10-20% of *de novo* AML (139) and up to 50% of cases of CMML (140). As one of the most prevalent mutations affecting hematopoiesis, the development of faithful murine models of *TET2* loss has been pursued by several research groups.

Multiple *Tet2* KO mouse models have been created, all demonstrating expansion of the HSC compartment following ablation of *Tet2* expression secondary to increased self-renewal capacity (113–117). In 2011, Moral-Crusio et al. developed a novel conditional *Tet2* KO murine model that resulted in MPD (113). This group utilized homologous recombination to introduce two *loxP* sites flanking exon 3 of *Tet2* in embryonic stem cells that were then injected into blastocysts. The resulting mice (*Tet2^fl/fl^*) were then crossed with transgenic *Vav*-Cre mice, resulting in *Tet2* deletion in the hematopoietic system *in utero*, leading to the development of a CMML-like disease by 20 weeks of life with monocyte–predominant leukocytosis and splenomegaly.

Another group developed a novel constitutive model *via* construction of a *Tet2*-targeting vector that disrupted the endogenous ATG start codon, leading to silencing of *Tet2* gene expression (116). The resultant mice with germline deletion of *Tet2* are viable and fertile but develop a CMML-like disease with leukocytosis, neutrophilia and monocytosis along with increased bone marrow cellularity, splenomegaly, and moderate liver enlargement. Approximately one-third of the *Tet2* ^-/-^ mice died within a year due to myeloid malignancy. These mice exhibited two distinct phenotypes: one with a population of erythroblasts infiltrating hematopoietic organs and the other with an aberrant population of mature myeloid cells including myeloblasts, monocytes/macrophages and neutrophils. Both malignant myeloid phenotypes caused massive hepatosplenomegaly and anemia.

These models of *Tet2* loss have proven useful for studies of collaborative leukemogenesis. A number of labs have conducted important studies combining *Tet2* deletion with other lesions that frequently co-occur in human AML, such as *Dnmt3a* as previously mentioned, *NRAS* and *KIT* (111, 118, 119). These models are crucial for clarifying the interplay between cooperative mutations providing models that closely mirror human disease.

### 5.3 *ASXL1*

Additional sex comb-like 1 (ASXL1) is a polycomb group protein that interacts with BAP1 and PRC2 to remodel chromatin thus regulate gene expression. *ASXL1* mutations have been reported in 5-11% of *de novo* AML (141–143). These mutations occur with increased frequency among older patients and are frequently detected in clonal hematopoiesis (142).

Abdel-Wahab et al. developed a novel murine model of *Asxl1* knockdown *via* retroviral transduction to introduce short hairpin RNA (shRNA) constructs into mouse bone marrow cells that were then transplanted into lethally irradiated recipient mice (144). They found that knockdown of *Asxl1* in a mouse expressing the oncogene *Nras^G12D^* accelerated the expected MPD, resulting in more severe symptoms of anemia and organomegaly as well as decreased lifespan. The same group then created a conditional KO model *via* insertion of two *loxP* sites flanking exons 5-10 of *Asxl1* (120). They crossed *Asxl1^fl/fl^* mice with transgenic mice harboring various tissue-specific Cre recombinase systems. *Ella-Cre^+^ Asxl^fl/fl^* mice had germline deletion of *Asxl1* and resulted in 100% embryonic lethality. Heterozygous germline deletion of *Asxl1* led to viable embryos at expected Mendelian ratios, though 35% of these mice exhibited craniofacial dysmorphism. Hematopoietic-specific deletion of *Asxl1* using either *Mx1-Cre* or *Vav-Cre* resulted in MDS with progressive anemia and leukopenia compared with littermate controls. They observed morphological dysplasia of peripheral myeloid cells and erythroid precursors with bone marrow hypocellularity.

A constitutive *Asxl1*-null murine model was created by Wang et al. the following year (121). This group replaced part of *Asxl1* exon 1 with *nlacZ/nGFP* reporter to disrupt the endogenous ATG and inhibit transcription of *Asxl1*. They used this technique to generate both *Asxl1* ^+/-^ and *Asxl1* ^-/-^ mice to compare the effects of haploinsufficiency versus complete loss of *Asxl1*. Complete loss of *Asxl1* resulted in an estimated 80% embryonic lethality, similar to the high rate observed in the inducible germline model that was previously published. Of the *Asxl1*-null mice, 80% that survived to birth died within the first day of life, and the remainder survived for 18-42 days. Surviving mice exhibited cytopenias and myeloid dysplasia, consistent with MDS-like disease. Interestingly, heterozygous deletion of *Asxl1* was also sufficient for development of a similar MDS-like disease. The disease phenotype became increasingly severe with age in *Asxl^+/-^* mice with worsening cytopenias and dysplasia, and progressed to a CMML-like disease in 22% (4 of 18 mice).

An alternative approach was taken to better mimic the truncating mutations that typically occur in human disease. A conditional knock-in mouse model was created by introducing a floxed mutant *Asxl1* allele *via* homologous recombination to mimic p.E635RfsX15 that is well-described in human disease (122). These mice were crossed with transgenic *Vav-Cre* mice in which recombination occurs in the hematopoietic system. Modest anemia was observed along with skewing of hematopoietic bone marrow population toward megakaryocyte progenitors and away from erythrocyte progenitors, but no frank MDS or AML was observed. However, when the authors incorporated concurrent expression of a mutant form of *RUNX1* into their mouse model, they found that co-expression of these two mutations led to development of frank MDS/AML with a short latency period following induced expression of the mutations (median survival of 160 days). They also performed retroviral insertional mutagenesis and found that all mice in this experimental cohort developed AML within a 1.5-year observation period.

Another knock-in model was recently created using CRISPR/Cas9 to introduce the most common MDS-associated mutation in humans, *ASXLl1^G643W^*, into murine embryonic stem cells resulting in expression of a truncated ASXL1 protein, mirroring that which occurs in humans (123, 145). This solo model again produced only mild impacts on hematopoiesis with skewing towards the myeloid lineage and mild splenomegaly. However, combination with an inducible model of *Cepba* haploinsufficiency drove the development of AML. Together these findings demonstrate the utility of a mouse model of *Asxl1* that recapitulates the pre-leukemic effect of this lesion and can be used to study its interaction with cooperating mutations in the progression to leukemia.

### 5.4 Cohesin Complex

The cohesin complex is a ring-like structure made up of various protein components including SMC1, SMC3, RAD21, and STAG1/2. Cohesin mediates the approximation of DNA fragments and plays a crucial role in sister chromatid cohesion, homologous recombination, and DNA looping. In turn, cohesin regulates gene expression *via* the approximation of distal enhancers and promoters. Somatic cohesin mutations have been identified in 12% of myeloid malignancies and often co-occur with genetic lesions such as t (8;21) and mutations in genes such as *TET2*, *NPM1*, and *ASXL1* (146, 147). Cohesin mutations are mutually exclusive, suggesting that reduction of one component leads to insufficiency of the entire complex. Therefore, the specific cohesin gene targeted in various murine models should not affect the disease. It is believed that cohesin’s vital role in sister chromatid cohesion makes complete cohesin loss incompatible with life. This hypothesis has been supported by the universally lethal effect of homozygous *Rad21* deletion in mice (148).

Two groups published mouse models of cohesin loss in 2015 using different genetic engineering techniques. Viny et al. (124) created a conditional knock-out model in which exon 4 is flanked by loxP sites. When crossed with transgenic *Mx1*-Cre mice, they induced complete loss of SMC3 in hematopoietic tissues *via* pIpC injections. Biallelic loss of *Smc3* resulted in 100% lethality within 11 days of Cre recombinase activation due to CNS hemorrhage and multiorgan failure. They then created a model of conditional *Smc3* haploinsufficiency *via* Cre-mediated deletion of a single *Smc3* allele in hematopoietic tissues and found a resultant increase in self-renewal capacity of HSCs. Alone, *Smc3* haploinsufficiency did not lead to phenotypic changes suggestive of MDS or leukemia, but the addition of *Flt3-*ITD expression within the mouse model of cohesin insufficiency led to AML with shortened latency compared to *Flt3-*ITD alone.

Alternatively, Mullenders et al. introduced shRNA to silence expression of cohesin components in a mouse model (125). They introduced a GFP transgene that housed a single copy of their engineered shRNA downstream of a tetracycline (Tet)-responsive element and crossed them with rtTA-transgenic animals to create an inducible and reversible model of cohesin knockdown. shRNAs were engineered to knockdown three of the core cohesin proteins, *Rad21*, *Smc1a* and *Stag2*. Mice were exposed to doxycycline starting at 6 weeks of age to induce shRNA expression. *Rad21* ^(shRNA/+)^, *Smc1a* ^(shRNA/+)^, *Stag2* ^(shRNA/+)^ mice all demonstrated a skewing toward the myeloid lineages. Interestingly, *Smc1a*-targeting shRNA expression resulted in a significant decrease in all core cohesin proteins rather than just in SMC1A. Over time, a subset of these cohesin knockdown mice developed a myeloproliferative neoplasm-like phenotype characterized by blood and bone marrow myeloid hyperplasia, hypocellular bone marrow, and splenomegaly.

shRNA knockdown and Cre recombinase-mediated deletion remain popular methods of modeling cohesin loss, particularly in novel models that combine cohesin insufficiency with other gene mutations to mirror leukemogenesis in humans. Both strategies effectively reduce gene expression and can be induced at specific timepoints to elucidate the precise order of acquisition that is hypothesized to occur in subsets of leukemia.

### 5.5 *BCOR*

*BCOR* is a tumor suppressor gene that encodes the transcription repressor BCL6 corepressor (BCOR). Over the past decade mutations in *BCOR* have been identified in 3.4-5% of cytogenetically normal AML in adults and 1.2% of pediatric AML (149–152). In 2017, Tanaka et al. created a transgenic mouse model using tamoxifen-inducible *Cre-ERT* in the ROSA26 locus to delete exon 4 of *Bcor* resulting in a truncated protein that lacks the BCL6 binding site (126). HSCs harvested from this model exhibited impaired repopulating capabilities. Half of their cohort developed T lymphoblastic leukemia but they did not observe development of myeloid malignancy.

Dr. Kelly and colleagues similarly generated a model in which exons 9 and 10 of the murine *Bcor* gene is flanked by *loxP* sites. When crossed to mice with transgenic *HSC-SCL-Cre*-*ER* ^T^ recombinase, Cre activation by tamoxifen injections lead to deletion of exons 9 and 10 of *Bcor* (termed *Bcor^ΔE9-10^*) specifically in HSCs, resulting in low level expression of a truncated protein that lacks the C-terminal PCGF Ub-like fold discriminator domain (127). *Bcor^ΔE9-10^* mice did not exhibit an abnormal hematopoietic phenotype but did demonstrate expansion of the myeloid progenitor compartment. The authors then found that combination of *Bcor^ΔE9-10^* and *Kras^G12D^* results in a highly penetrant model of myeloid leukemia with significantly decreased survival compared to leukemia resulting from *Kras^G12D^* alone.

Findings from recent *Bcor*-mutated murine models of AML have established a strong role for *Bcor* in the development of acute erythroid leukemia (AEL). Drs. Brunangelo Falini, Margaret Goodell, and colleagues collaborated to generate a double *Dnmt3a* ^-/-^ and *Bcor* ^-/-^ mouse model (128). They first created conditional *Bcor* knockout in which exons 8-10 are flanked by *loxP* sites crossed with transgenic *Mx1-Cre* to induce *Bcor* knock out in the hematopoietic system. These mice were then mated with others carrying two floxed *Dnmt3a* alleles and induced full knockout of both genes, which resulted in a fully penetrant and lethal AEL. They then used this model to demonstrate the efficacy of the demethylating agent decitabine, which exhibited better control of tumor burden than cytarabine alone. Dr. Charles Mullighan and colleagues used multiplex genome editing in HSPCs that were transplanted into primary recipient mice and similarly found that co-occurrence of mutations in *Bcor* and *Trp53* strongly promoted development of AEL (76).

These existing murine models recapitulate what is observed in humans and will help to better understand the precise role of *BCOR* loss as it cooperates with leukemogenic mutations in other cellular pathways as well as the role it plays in therapeutic response.

## 6 Other Recurrent AML Mutations

There are several recurrently mutated genes that do not fall into the specific categories discussed above which have distinct roles in leukemia pathophysiology. These mutations will be reviewed below.

### 6.1 *NPM1*

Mutations of the gene *NPM1* are among the most common mutations in adult AML, found in up to 35% of patients (15). While less common in childhood AML (8-10%), like in adults, *NPM1*-mutant AML is generally associated with a favorable prognosis, co-occurrence with *FLT3*-ITD mutations, and *HOX* gene overexpression (18). NPM1 is a molecular chaperone with roles in centrosome duplication, ribosome biogenesis, and stress-induced regulation of P53 (153). In its wild-type state, NPM1 shuttles rapidly between the nucleus and cytoplasm, and predominantly localizes to the nucleoli. In AML, frameshift mutations of *NPM1* lead to its aberrant cytoplasmic localization, thus the common designation, NPMc+ (15). Given the prevalence of *NPM1* mutations in AML, there has been much interest in generating murine models with which to study the biology of *NPM1*-mutant AML.

In 2010, researchers from the lab of Dr. Pier Paolo Pandolfi published their work developing and characterizing a murine model in which mutant *NPM1* was transgenically expressed under the control of the *hMRP8* promoter, resulting in overexpression of mutant NPM1 in myeloid progenitors. These mice developed myeloproliferation in the bone marrow and spleens but did not develop overt AML (154). This model was later crossed to mice with a heterozygous germline *Flt3*-ITD mutation, demonstrating cooperativity between these commonly co-occurring lesions (98). In 2012, another group developed a model in which they knocked-in TCTG into murine *Npm1* exon 11, similar to the most common frameshift mutation found in human AML. While the resultant mutant protein differed slightly from human NPMc+ protein, the group still found excess cytoplasmic expression. This strategy led to an incompletely penetrant MPD, but like the transgenic model from the Pandolfi group, did not develop AML (155). Around this same time, Vassiliou et al. developed the first conditional NPMc+ model, by knocking-in a ‘humanized’ exon 11 frameshift mutation (the most common mutation in AML) just downstream of murine exon 11. They also knocked-in *loxP* sites flanking murine exon 11, such that Cre-mediated recombination leads to excision of the native exon 11 and exclusive expression of humanized mutant *Npm1c*. As expected, when induced in the hematopoietic system by pIpC activation of *Mx1*-Cre, the mutant protein localized predominantly to the cytoplasm. Expression of mutant NPM1c led to Hox gene overexpression, enhanced stem cell self-renewal and myeloid skewing with ~1/3 of the mice developing a delayed onset AML (156). When crossed to mice constitutively expressing *Flt3*-ITD mutation, mice with induced *Npm1c* expression developed a rapid onset AML, confirming molecular synergy (157). This model has also proved useful for pre-clinical investigations including studies demonstrating the therapeutic potential of inhibitors of the MENIN-MLL interaction for the treatment of NPMc+ AML (158).

More recently, the lab of Dr. Jennifer Trowbridge designed a humanized, inducible *Npm1c* mutation similar to that developed by Vassiliou et al., but instead of a Cre-inducible system, utilized an Flp-recombinase inducible system. They crossed these mice with transgenic mice in which the tamoxifen-inducible FlpoER is knocked into the *Gt* (*ROSA) 26Sor* locus. This strategy allowed for the sequential induction of both a Cre-inducible *Dnmt3a^R878^* mutation (see DNMT3A section above) and the FLP-inducible expression of *Npm1c*. A potential disadvantage of this strategy is that because *FlpoER* is knocked into the *Rosa26* locus it is expressed ubiquitously; to isolate the hematologic effects of these combined mutations, hematopoietic transplantation was necessary. In isolation, induction of the *Npm1c* mutation in the hematopoietic system led to a low penetrance MPD but no overt AML. However, when induction of *Npm1c* was preceded by induction of the *Dnmt3a* mutation, the mice developed a highly penetrant MDS and/or MPD. Interestingly, the disease latency was inversely correlated with the length of time between induction of the *Dnmt3a* mutation and the *Npm1c* mutation (i.e., the longer the mouse had expression of mutant *Dnmt3a* prior to induction of *Npm1c* expression, the shorter the disease latency) (110).

### 6.2 *TP53*

Greater than 50% of human cancers carry mutations in the well-established tumor suppressor gene *TP53* (159). Interestingly *TP53* mutations are only detected in less than 10% of *de novo* AML but confer high risk disease with an extremely poor prognosis (10, 160). Creation of a mouse model of *TP53*-mutant AML is important for further therapeutic development in this traditionally hard to treat subset. Isolated mutations in *Trp53* have proven insufficient to induce leukemogenesis in mouse models but can be combined with commonly co-occurring mutations to generate models of myeloid leukemia that can be used to define the role of cooperative mutations and provide an environment for the development of new therapeutic targets and strategies.

Several transgenic mouse models have shown that *Trp53* cooperates with other genetic aberrations to hasten the development of AML. In the development of these AML models it was important to utilize non-germline methods to reduce p53 expression as *Trp53* null mice are prone to developing T cell malignancies. Stoddart et al. created a novel model of therapy-related AML using a *Trp53*-targeting shRNA to knock down expression of p53 in bone marrow cells harvested from transgenic mice harboring heterozygous loss of *Erg1* and *Apc*, two genes located on the long arm of chromosome 5, and thus lost in AML with 5q deletion (161). While they were successful in generating a novel model that may mirror the behavior of therapy-related AML, the low penetrance of disease (17%) likely indicates that additional genes lost with 5q deletion play an important role in the transformation to AML. This is supported by work done by Yang et al., who recently generated dual transgenic mice with Gilliland *Flt3*-ITD knock-in model and either heterozygous or homozygous *Trp53* deletion (162). Heterozygous *Trp53* knockout significantly increased the penetrance and lethality of myeloid leukemia compared to *Flt3*-ITD alone, and homozygous *Trp53* knockout led to a further decrease in median survival.

Additional models have been created to recapitulate the common co-occurrence of mutations in *TP53* and Ras family genes and determine their cooperative roles in leukemogenesis. *Zhou* et al. found that *Trp53* depletion accelerated AML development in mice expressing *Kras^G12D^* (163). Members of Dr. Jing Zhang’s lab then created a conditional transgenic model of AML using *Mx1*-Cre to induce homozygous loss of *Trp53* in the hematopoietic system and concurrently induce expression of the oncogenic *Nras^G12D^* (164). They used RNA sequencing to define the transcriptome of the leukemic cells and determined that *Trp53* loss cooperated with mutant *Nras* within the megakaryocyte-erythroid progenitors to result in AML.

Together, these findings have shown the utility of generating mouse models of *Trp53*-mutant AML to advance our understanding of and develop new treatment strategies for this aggressive subcategory of AML.

## 7 Patient Derived Xenografts

The murine models of AML discussed above are essential tools to study leukemia development and unfold molecular mechanisms related to the disease. Despite sophisticated advancements in transgenic mouse modeling, there are differences in the murine and human leukemic phenotype that require humanized models to better understand nuances of human disease. *Ex vivo* models with human AML blasts have been developed for these purposes but are limited due to the missing interaction of the human AML blasts with the bone marrow microenvironment which plays an important role in leukemia development and therapeutic response. Here we review patient derived xenograft models that have been developed and improved over time to overcome these limitations.

The development of immunocompromised mouse models was essential for the study of patient-derived tumors and preclinical discovery of new compounds for cancer treatment (**Table 4**). The first immunodeficient mouse model was described in 1966 by Flanagan (165). These nude athymic (nu/nu) mice were T-cell-deficient due to a homozygous *Foxn1* mutation and enabled the study of human cancer in mice (166). However, this model has its limitations for studying AML since the intact murine B- and natural killer (NK)-cell populations lead to poor human AML engraftment (167). The severe combined immune deficiency (SCID) phenotype was first described in C.B-17 mice in 1983, caused by a homozygous mutation in the *Scid* gene (scid/scid). The SCID immunodeficient mouse model proved superior to nude mice for *in vivo* studies of human cancers (168). The *Scid* mutation affects the VDJ recombinase system and results in a lack of mature T- and B-lymphocytes. In the early 1990s, multiple groups showed the engraftment of human myeloid leukemia cell lines and patient AML cells in SCID mice, indicating that this PDX model is a useful tool for studying AML. However, the SCID model still had technical limitations as some human AMLs failed to engraft without exogenous cytokine treatment (169–171). Reports by Carrol et al. and Riggs et al. suggested that this mouse model is “leaky” as mice showed the presence of mature lymphocytes, likely explaining the relative engraftment barrier (182, 183).

**Table 4:** Mouse models available for patient-derived xenografts of AML.

Mouse Strain	Common Abbreviation	Immune system	Cytokines expressed	Engraftment of AML			References
				High-Risk	Intermediate Risk	FavorableRisk	
**nude athymic**	nude	T-cell deficient	–	–	–	–	(165–167)
**C.B-17 SCID**	SCID	no functional T- and B-cells	–	+	–	–	(168–171)
**NOD/LtSz-scid/scid**	NOD/SCID	T- and B-cell deficient, reduced NK and macrophage activity	–	++	–	–	(172–175)
**NOD-scid Il2rγ^null^**	NSG	no functional T-, B- and NK cells	–	+++	++	+	(176)
**NSG–SGM3**	NSGS	no functional T-, B- and NK cells	hIL-3, hGM-CSF, hCSF	++++	++++	++	(177, 178)
**MISTRG**	–	no functional T-, B- and NK cells	hM-CSF, hIL-3, hGM-CSF, hTPO	++++	++++	+++	(179–181)

Throughout the years more severe immunodeficient mouse models have been developed to improve engraftment, including the non-obese diabetic NOD/SCID (NOD/LtSz-scid/scid) mouse. SCID mice were backcrossed onto the NOD/Lt strain background resulting in a strain that is deficient in B- and T-lymphocytes and shows reduced NK-cell and macrophage activity (172). In contrast to SCID mice, normal human bone marrow cells and patient-derived AML cells can be engrafted with much higher efficiency in NOD/SCID mice using fewer cells (173, 174, 184, 185). A robust engraftment of primitive CD34^+^CD38^-^ leukemic progenitors, also known as NOD/SCID leukemia-initiating cells (NOD/SL-IC) or leukemic stem cells (LSC), was observed in NOD/SCID mice. Serial transplantation of LSCs and their potential to differentiate into leukemic blasts provides the opportunity to study those leukemia initiating clones more closely and identify potential drugs against them. However, while the NOD/SCID xenograft model has shown efficient engraftment for high-risk AML cases, it is still limited by poor engraftment for favorable and intermediate-risk AML (175).

The introduction of a deletion of the *interleukin 2 (IL2) receptor gamma chain* (*Il2rγ*) gene led to an even more immune compromised mouse model, the NOD-scid Il2rγnull (NSG) mouse, that improved engraftment of patient AML cells regardless of the French-American-British classification or cytogenetic features (176). Better engraftment and longer lifespan (>16 months) made NSG mice the preferred immunodeficient model for AML research over NOD/SCID mice. Studies in NSG mice showed that human LSCs can home to the bone marrow niche of the mouse resulting in quiescent LSC that are resistant to chemotherapy (186). Those features of the NSG PDX model that recapitulates human AML facilitate the study of chemotherapy resistance conferring mechanisms. The use of NSG PDX models has also led to a better understanding of the heterogeneity of leukemia-initiating cells (LIC) as reports identified the existence of not only CD34^+^CD38^-^ LIC cells but also CD34^+^CD38^+^ and even CD34^-^ populations capable of initiating AML (187, 188).

Over time more strains have been developed to overcome the limitations of the previous models. Wunderlich et al. generated a mouse strain that transgenically expresses the human cytokines stem cell factor (SCF), GM-CSF, and IL-3 (SGM3) in the NOD/SCID background (177). Usage of NOD/LtSz-scid IL2RG–SGM3 mice (NSGS) led to superior engraftment of AML cells compared to other strains (177, 178). Even AML samples from favorable risk groups like CBF AML, *NPM1-*mutated/*FLT3* wild type, or *CEBPA-*mutated AML, that are difficult to engraft in previous immunocompromised strains, were capable of engrafting in NSGS mice (177, 178).

An even more advanced immunodeficient mouse strain was developed by Dr. Flavell and colleagues (179). MISTRG mice are Rag2^-^ and IL2rγ-deficient, with genes for four human cytokines (hM-CSF, hIL-3, hGM-CSF, and hTPO) knocked-in to their respective mouse loci. Because they are under the control of their endogenous promoters, they are expressed at physiologic levels as opposed to the supraphysiologic levels in NSGS mice. The MISTRG mice also express human SIRPa which binds to human CD47 and results in inhibition of phagocytosis of human cells and hence supports better engraftment (180). Ellegast et al. reported successful engraftment of *NPM1* mutated and inv (16) AML samples using this advanced mouse model (181). They also reported an important role for M-CSF expression in mice using MSTRG (knock-in for only hMCSF and hTPO) as an important factor for inv (16) AML engraftment. The successful engraftment of t (8;21) AML cells in MISTRG mice that leads to leukemia has not yet been shown.

The development of new immune compromised mouse models over the last few decades has led to increasingly efficient engraftment of human AML samples. This gave rise to new insights into AML hierarchy, genetic and functional characteristics of human AML, and the option for drug testing on human AML cells in an *in vivo* model. Yet, there are still challenges that need to be addressed in the future such as efficient engraftment of less aggressive AML subtypes. Recently, it has been shown by Reinisch et al. that establishing a human bone marrow niche in the mouse by transplanting human bone marrow-mesenchymal stem cell (MSC)-derived ossicles results in robust and superior engraftment of human AML samples (189). In another study, hMSC were seeded in a gelatin-based porous scaffold and cultured *in vitro* (190). Before implanting the scaffolds into NSG mice, patient-derived AML cells were pre-seeded into the scaffold. This method led to successful engraftment of AML samples that failed to engraft in NSG mice after intravenous injection of the leukemic cells. This approach has also been tested for other stromal cell types like endothelial cells and osteoblasts. Since there is crosstalk between leukemia cells and the bone marrow niche, these kinds of models help to study the leukemia bone marrow microenvironment and possible cell-extrinsic mechanisms that may contribute to chemotherapy resistance.

Another xenograft mouse model to study leukemia has been developed using isolated human CD34+ hematopoietic cells from healthy donors that are transduced with a viral vector expressing system or have been genetically engineered which are then transplanted into immunodeficient mice. Wei et al. used the retroviral transduction method to express the KMT2A-MLLT3 fusion protein in human CD34+ cord blood (huCB) cells resulting in indefinite proliferation of the cells *in vivo* and *in vitro* (191). Transplanting the KMT2A-MLLT3 expressing cells into different immunodeficient mouse models led to AML in NSGS mice and AML, B-ALL or mixed lineage leukemia in NSG and NSG beta 2 microglobulin (NS-B2M) mice showing that the immune microenvironment plays an important role in leukemia development. In this approach both copies of the wild-type *KMT2A* gene are present which is not found in *KMT2A*r leukemias resulting in unknown regulatory effects by the wild-type protein. Dr. Corinna Buechele and colleagues used transcription activator-like effector nucleases (TALENs) gene editing tool to introduce DNA double strand breaks at known breakpoint cluster regions in the *KMT2A* gene and nucleofected with DNA templates with KMT2A homology flanking the sites targeted by TALENs, fusion partner (*MLLT3 or MLLT1*) cDNA, and a fluorescent tag. The strategy resulting in the expression of the designed KMT2A fusion proteins in huCB cells (192). The model was sufficient to initiate leukemia in NSG mice and recapitulated many clinical features of *KMT2A*r leukemias. A model for AML with t(6;9)(p22;q34) was successfully established by transplanting human CD34+ cells transduced with the *DEK-NUP214* fusion gene into NSGS mice resulting in AML development and showing phenotypic and genetic features of human t(6;9) AML (193). Genetic alteration of human CD34+ hematopoietic cells and transplantation into immunodeficient mice provides a humanized mouse model to study the leukemic potential of mutations and chromosomal aberrations found in AML without the potential confounding effects of the co-occurring mutations inherent to human AML cell lines and primary patient samples.

## 8 Conclusion

For AML, like many other human diseases, mouse models are indispensable research tools. Genetically engineered models of common AML driver lesions have provided invaluable insight into disease mechanisms, led to identification of therapeutic targets, and enhanced our understanding of the intricate interactions between collaborating genetic and epigenetic events. Engraftment of human AML cells into immunocompromised mice has expanded our understanding of tumor heterogeneity, shaped the definition of the leukemia initiating cell and allowed for critical pre-clinical investigations of promising novel therapeutics.

Over just the last few years, research employing murine models of AML has resulted in several highly significant advancements in the field. For example, we have learned that not just the combination of mutations matter in the genesis of AML, but the order of mutation acquisition, the age at which the mutations are acquired, and even the length of time between collaborating mutations critically impacts disease development (40, 118). Additionally, efforts to develop mouse models of rare genetic disease entities, such as *NUP98*-rearranged and *BCOR* mutant AML have shed new mechanistic light on previously poorly understood genetic drivers and revealed novel therapeutic vulnerabilities (51, 52, 75, 77, 128). Further, ongoing improvements in immunodeficient mouse strains coupled with efficient genome engineering tools have allowed for not only the engraftment of virtually all subsets of human AML cells into mice, but also for the transformation of normal human hematopoietic cells into leukemic cells capable of engraftment into immunocompromised mice (179, 189, 191–193).

While undeniably powerful, limitations of murine models still must be acknowledged including potential differences in murine and human biology, differences in the malignancies that develop in mice compared to humans, and remaining challenges in engraftment of certain human leukemias into immunodeficient murine recipients. However, with numerous labs around the world working to optimize existing and generate new and relevant murine models, the field will undoubtedly continue to advance, paving the way for ongoing improvements in the care of patients with AML.

## Author Contributions

RR, SC, KK and MO’K participated in the conception and content planning for the review. KK and SC contributed equally to the composition of the manuscript. MO’K, KW and RR contributed substantially to the fabrication of the paper. RR reviewed and edited the manuscript as last author. All authors contributed to the article and approved the submitted version.

## Funding

KK was supported by CPRIT RP210027 - Baylor College of Medicine Comprehensive Cancer Training Program. SC was supported by the St. Baldrick’s Foundation and Children’s Cancer Research Fund.

## Conflict of Interest

The authors declare that the research was conducted in the absence of any commercial or financial relationships that could be construed as a potential conflict of interest.

## Publisher’s Note

All claims expressed in this article are solely those of the authors and do not necessarily represent those of their affiliated organizations, or those of the publisher, the editors and the reviewers. Any product that may be evaluated in this article, or claim that may be made by its manufacturer, is not guaranteed or endorsed by the publisher.
