# Corrigendum: Murine models of acute myeloid leukemia

**DOI:** 10.3389/fonc.2022.1089874

**Published:** 2023-02-23

**Authors:** Kristen J. Kurtz, Shannon E. Conneely, Madeleine O’Keefe, Katharina Wohlan, Rachel E. Rau

**Affiliations:** ^1^ Department of Pediatrics, Baylor College of Medicine, Texas Children’s Hospital, Houston, TX, United States; ^2^ Department of Molecular and Cellular Biology, Baylor College of Medicine, Houston, TX, United States

**Keywords:** acute myeloid leukemia, AML, transgenic mouse, genetically engineered mice (GEM), core binding factor acute myeloid leukemia, KMT2a (MLL) rearrangements, NUP98 fusion, patient-derived xenograft (PDX)

In the original article, there was an error in **Table 1** as published. We inadvertently omitted a CBFA2T3-GLIS2 mouse model and two NUP98 fusion models. The corrected **Table 1** and its caption appear below.

**Table 1:** Mouse models of fusion genes.

Fusion Gene	Year	Expression	Mechanism	Phenotype	References
*RUNX1-RUNX1T1*	2006	Constitutive	Retrovirus	9a isoform: AML	(37)
	2001	Constitutive	Germline - *Mrp8* promoter (myeloid specific)	AML, T-ALL after ENU treatment	(38)
	2013	Inducible		MDS	(39)
	2021	Inducible	Tet-On eR1-CreER^T2^	AML, MPD	(40)
*CBFB-MYH11*	2006	Inducible	Mx1-Cre	AML	(41)
***KMT2A-MLLT3***	1996	Constitutive	Germline	AML	(42)
	2000	Inducible	Lmo2-Cre	AML	(43)
	2013	Constitutive	Retrovirus	AML	(44)
	2016	Inducible	Retrovirus	(Transduced LSKs > GMP) AML	(45)
*MLL-*PTD	2012	Constitutive	Germline	AML (if *FLT3*-ITD mutated)	(46)
***KMT2A-MLLT1***	2013	Inducible	CreER	ALL	(47)
	2014	Inducible	Tet-On	ALL	(48)
*NUP98-NSD1*	2007	Constitutive	Retrovirus	AML	(49)
	2014	Constitutive	Retrovirus	AML (if *FLT3*-ITD mutated)	(50)
	2020	Constitutive	Retrovirus	AML	(51)
Other *NUP98* fusions	2020	Inducible	Tet-On Retrovirus	AML	(52)
*CBFA2T3-GLIS2*	2019	Inducible	Tet-On	AML	(53)
*PML-RARA*	1997	Constitutive	Germline – *Ctsg* promoter (myeloid specific)	AML – long latency	(54)
	1999	Constitutive	Retrovirus	Differentiation blockade, enhanced self-renewal	(55)
	2003	Constitutive	Germline – *Mrp8* promoter (myeloid specific)	AML	(56)

AML, acute myeloid leukemia; ENU, N-ethyl-N-nitrosurea; GMP, granulocyte-macrophage progenitor; ITD, internal tandem duplication; LSK, Lin^-^Sca1^+^Kit^+^; MDS, myelodysplastic syndrome; MPD, myeloproliferative disease; PTD, partial tandem duplication; T-ALL, T-acute lymphoblastic leukemia.

**Text Correction**

In the original article, several sections were mislabeled. The sub-section titled “Core binding factor leukemias” currently is labeled with “4”. This has been corrected to 3.1.

The sub-section titled “NUP98 Fusions” currently is labeled as section 3.4. This has been corrected to 3.3.

**Missing reference**

In the sub-section “NUP98 Fusions”, we failed to reference an important paper, Wang et al., 2007.

We have therefore inserted a new sentence and modified a sentence in that section.

Original text: Retroviral models of *NUP98* fusions have served as the predominant mouse models to date. Mohanty et al. showed that retroviral model of *NUP98-NSD1*, the most common *NUP98* fusion in pediatric AML, into murein HSCs followed by transplantation independently produced AML with a median survival of 250 days post-transplant with significant disease acceleration when *NRAS* ^G12D^ is co-expressed.

Corrected text:

Retroviral models of *NUP98* fusions have served as the predominant mouse models to date. In 2007 Wang et al. showed that retroviral transduction of *NUP98-NSD1*, the most common *NUP98* fusion in pediatric AML, into murine hematopoietic progenitors followed by transplantation independently produced AML with an average survival of 126 days post-transplant (68). Mohanty et al. created a similar retroviral model of *NUP98-NSD1* and demonstrated significant disease acceleration when *NRAS^G12D^* is co-expressed.

**Text correction**

In the sub-section, “CBFA2T3-GLIS2” we originally did not include an important mouse model reported by Lopez, et al. The corrected text appears below.

Lopez et al. (53) developed a novel transgenic model using tetracycline-inducible expression of *CBFA2T3-GLIS2* in which nearly all mice developed a lethal hematologic malignancy, 20% of which displayed megakaryoblastic markers with a disease latency of 164 days, whereas the remaining mice displayed heterogeneous non-megakaryoblastic immunophenotypes with a significantly longer disease latency (79). As this fusion is predominantly found in pediatric AML, the authors sought to investigate the role of developmental stage on leukemia onset. They found that transplantation of fetal liver cells from their inducible model led to a shorter disease latency with a predominant megakaryoblastic population compared to mice transplanted with bone marrow from adult mice, demonstrating that developmental stage significantly impacts disease phenotype. The Lopez et al. transgenic model was a substantial technical advancement as prior to its description, murine models had been largely limited to patient-derived xenografts (PDXs) (80).

The authors apologize for these errors and state that they do not change the scientific conclusions of the article in any way. The original article has been updated.

